# Insulitis in human type 1 diabetes: a comparison between patients and animal models

**DOI:** 10.1007/s00281-014-0438-4

**Published:** 2014-07-09

**Authors:** Peter In’t Veld

**Affiliations:** Department of Pathology, Diabetes Research Center, Vrije Universiteit Brussel (VUB), Laarbeeklaan 103, 1090 Brussels, Belgium

**Keywords:** Autoimmunity, Insulin, Insulitis, Islets of Langerhans, Pancreas, Type 1 diabetes

## Abstract

Human type 1 diabetes (T1D) is considered to be an autoimmune disease, with CD8+ T-cell-mediated cytotoxicity being directed against the insulin-producing beta cells, leading to a gradual decrease in beta cell mass and the development of chronic hyperglycemia. The histopathologically defining lesion in recent-onset T1D patients is insulitis, a relatively subtle leucocytic infiltration present in approximately 10 % of the islets of Langerhans from children with recent-onset (<1 year) disease. Due to the transient nature of the infiltrate, its heterogeneous distribution in the pancreas and the nature of the patient population, material for research is extremely rare and limited to a cumulative total of approximately 150 cases collected over the past century. Most studies on the etiopathogenesis of T1D have therefore focused on the non-obese diabetic (NOD) mouse model, which shares many genetic and immunological disease characteristics with human T1D, although its islet histopathology is remarkably different. In view of these differences and in view of the limited success of clinical immune interventions based on observations in the NOD mouse, there is a renewed focus on studying the pathogenetic process in patient material.

## Human type 1 diabetes

Type 1 diabetes (T1D) is a chronic (auto)immune disease that causes a specific destruction of most insulin-producing pancreatic beta cells, leading to overt hyperglycemia, a need for lifelong exogenous insulin replacement therapy and a high risk for developing debilitating chronic complications. Although the disease can occur at any age, there is a peak in newly diagnosed cases between 5 and 7 years of age and at or near puberty. In contrast to other autoimmune diseases, where there is a clear female preponderance, slightly more males develop T1D than females. The incidence shows geographical variability, ranging between 0.1 and 60 cases per 100,000 people, but its true level is difficult to ascertain as the disease is probably under-diagnosed, with many T2D patients also showing signs of immunological involvement [1].

The classical model for the pathogenesis of T1D is that an environmental factor triggers autoimmunity in genetically susceptible individuals. As discussed below autoantibodies against insulin develop first, followed by other types of autoantibodies, often directed against components of the insulin secretory granule. The presence of autoantibodies is usually taken as a sign of ongoing beta cell destruction, with beta cell mass decreasing as autoimmunity expands as witnessed by the increasing number of autoantibodies. The decrease first becomes evident as signs of glucose intolerance develop, followed by clinical diabetes when a threshold of residual beta cell mass is reached that is usually suggested to be around 10 % of normal [2]. As will be shown, many elements of this classical model are currently under debate.

This review deals specifically with insulitis, a multifocal inflammatory reaction limited to the islets of Langerhans considered to be characteristic for T1D and responsible for the severe loss of insulin-producing beta cells, resulting in loss of glycemic control and its clinical consequences. It summarizes the main findings in patients and discusses the similarities and differences of the human disease with a frequently used animal model for the disease, the non-obese diabetic (NOD) mouse. The genetics, immunology and clinical aspects of the disease have been discussed in many excellent reviews [1, 3–5] and will only be discussed briefly.

Progress in establishing the etiopathogenesis of T1D has been slow. There are several reasons for this: (a) the diffuse nature of the endocrine pancreas makes it a difficult object to study. The endocrine islets of Langerhans are scattered throughout the exocrine parenchyma and form only 1–2 % of gland volume [6]. The focal nature of some of the histopathological changes, with inflammatory lesions observed in some pancreatic lobes but not in others, requires multiple samples for study. In addition, some of the lesions, like insulitis, are transient and usually only observed in recent-onset patients. (b) Pancreatic biopsies are not normally taken in T1D patients due to the risk of pancreatitis. Biopsies have only been used in small series of recent-onset T1D patients [7]. Although initially considered to be relatively safe [8], they were later shown to have considerable side-effects and recent clinical trials were ended prematurely. Very few T1D patients have therefore been biopsied. (c) Due to the nature of the pancreatic gland, with its high content of digestive enzymes, the tissue is prone to rapid autolysis and well-preserved tissue is often only obtained from patients who were autopsied within a few hours after death. (d) Improved clinical management has resulted in fewer patients dying in diabetic ketoacidosis, leading to a decreased number of cases coming to autopsy. Together, these factors make that very few cases are available for study. The most characteristic lesion in T1D, the presence of an inflammatory infiltration of the islets of Langerhans (insulitis), has been reported in cumulative total of only 150 cases collected over the past 100 years [9]. Most of these cases are no longer available for study; detailed clinical information is often lacking and the material is usually fixed and paraffin-embedded, which limits molecular analysis. In practice, this means that few pathologists have ever seen insulitis during their professional career and that each year on average only one or two new recent-onset T1D cases become available for study worldwide.

The lack of human material has led many investigators to focus on animal models for the disease. In this context, the NOD mouse has become the model of choice. The NOD mouse shares many characteristics with human T1D, but as will be discussed below, there are also considerable differences. Although animal models yield invaluable information on molecular and cellular processes under conditions of autoimmunity, they also carry the risk that they may not represent the human disease in all its complexity, risking a situation where our view of disease progression is to a large extent derived from animal studies rather than from observations in patients. Careful and continuous validation of the animal model is therefore necessary to mitigate such a risk. Differences between the NOD mouse model and T1D patients have been detailed in a number of recent overviews [10–12], and concern has been raised that the differences in etiopathogenesis might be more profound than previously realized. Such concerns have been amplified by the fact that many immune intervention studies, often based on often dramatically positive results in NOD mice, resulted in limited success in patients [13].

Many potential treatments have been developed in the NOD mouse by which the disease can be postponed, prevented, or even cured after onset of overt symptoms. These interventions were tabulated in a number of reviews in which it was noted that >195 immune interventions were successful in the NOD model, but that virtually none were effective in patients [11, 12]. One immune intervention developed in the NOD mouse model, consisting of a series of low dose anti-CD3 antibody injections [14], was found to transiently preserve residual beta cell function in patients when given shortly after disease onset [15]. However, the effects are minor compared to those in the NOD mice, where the same treatment leads to complete remission.

Due to these somewhat disappointing results, there is a renewed interest in islet pathology in human material in order to validate previous observations made in the NOD mouse and to study in more detail any differences that are established. Such new research is facilitated by the recent establishment of large tissue banks with optimally preserved human donor pancreas obtained from control, sub-clinical T1D or recent-onset T1D subjects [9, 16].

### The discovery of insulitis

Insulitis is defined as a predominantly lymphocytic infiltration, limited to the islets of Langerhans. It was first described in 1902 in a 10-year-old who died in ketoacidosis [17] and was termed ‘insulitis’ by the Swiss pathologist von Meyenburg [18]. Although initially considered to be a rare event, it was later found to be characteristic for diabetes in children with recent-onset (<1 year) disease [19, 20]. The infiltrate contains predominantly CD8+ lymphocytes, in addition to CD4+ lymphocytes, B lymphocytes and macrophages [21]. Cellular immunity is accompanied by humoral immunity, with circulating autoantibodies against several different beta cell autoantigens [1]. The nature of the autoantigen against which the immune response is directed is still being debated, as will be discussed below.

Although insulitis was first observed more than a century ago [17], its true significance was not recognized until 1958 when Lecompte [20] observed that the lesion appeared to be characteristic for children with acute onset disease and short duration. He stressed that the lesion was relatively rare, but that it might be under-diagnosed as it is easily missed using the conventional stains then in use. In 1965, Gepts [19] was able to assess the incidence of insulitis in a large cohort of patients: he studied 22 recent-onset cases (<1 year) of diabetes below the age of 40 and found that 15 cases (68 %) showed insulitis. He also observed that the insulitic lesions appeared to be transient as they were no longer present in patients with a disease duration of >1 year. Lastly, he showed that beta cell mass in his study group was reduced to approximately 10 % of normal controls and that many islets were atrophic, consisting only of non-beta cells. Arguably, he laid the basis for the classification of T1D as an autoimmune disease [22]. In a 1978 follow-up study [23], using the then newly developed immunohistochemical staining techniques for islet hormones, he observed that insulitis preferentially targeted islets with remaining beta cells and that it was virtually absent in islets from which the beta cells had disappeared. This led him to propose that ‘insulitis represents an immune reaction of the delayed type, specifically directed against beta cells’.

His studies were confirmed by Foulis [24], who used a computerized survey of deaths in the UK and was able to retrieve tissue blocks of 119 patients who died in diabetic ketoacidosis before the age of 20. He observed insulitis in 47 out of 60 patients with recent-onset (<1 year) disease (78 %). He observed that insulitis was present in 23 % of the insulin-containing islets, and in 1 % of the insulin-deficient islets, thus confirming Gepts’s observation that the immunological reactivity appeared to be directed specifically to beta cells.

Together, the studies by Gepts and Foulis represent almost half of the 150 cases with insulitis that have been described up to this day [7, 8, 17–20, 23–54]. Major follow-up studies have been lacking due to the reasons outlined above, and only recently a renewed effort to study insulitis in human T1D was initiated using large biobanks of human donor pancreas in Brussels and Gainesville-FL (JDRF-nPOD).

### Phenotyping insulitis

The infiltrating cells in insulitic lesions were first phenotyped in a 12-year-old girl who died 1 month after diagnosis [26]. Immunohistochemical staining showed that these cells consisted mainly of T lymphocytes, with the T-cytotoxic/suppressor subtype being most abundant, and T-helper cells and other leucocytes being present at lower frequency. Later studies confirmed a predominantly CD8+ T-cell phenotype but also pointed to an important presence of macrophages [35, 42]. An extensive reanalysis of 279 individual islets with insulitis from 29 recent-onset cases showed that the composition differed depending on the stage of insulitic lesion: early stage lesions, where beta cell loss is not yet evident, showed infiltration of predominantly CD8+ T cells in addition to CD68+ macrophages, CD4+ T lymphocytes and CD20+ B lymphocytes. late-stage insulitic lesions, with <10 % beta cells in the islets, showed a similar composition, with the exception of CD20+ B lymphocytes that were four-fold more frequent than in early stage lesions. Tetramer staining of HLA-0201-positive patients [29] with a disease duration of <1 week to 8 years showed that CD8+ T cells found within insulitic lesions recognize several different islet antigens including amino acid sequences from insulin, preproinsulin (aa 15–23), islet-specific glucose-6-phosphatase catalytic subunit related protein (IGRP, aa 265–273), IA-2 (tyrosine phosphatase-like protein aa 797–805), GAD65 (glutamic acid decarboxylase aa 114–123) and preproIAPP (human islet amyloid polypeptide precursor protein aa 5–13). The four recent-onset patients that were tested showed only islets with a single positivity for islet-autoreactive T cells (insulin or IGRP), indicating a possible clonal origin of the infiltrating T cells, while four chronic patients with disease duration longer than a year showed multiple islet-reactive specificities. These results are consistent with an autoimmune CD8 T-cell-mediated beta cell loss that is initially directed against insulin (and or IGRP) epitopes and later, because of the release of additional beta cell antigens caused by ongoing beta cell death, evolves into a broader reactivity pattern. The (tentative) identification of T-cell specificities opens new strategies for treatment, possibly via the induction of tolerance. Clearly, studies into the specificity of the infiltrating CD8 T cells are still in an early phase. The number of patients that were investigated was small, they were already diagnosed with the disease, and as the authors stress themselves, the signal intensity of the tetramer staining was very low. Interestingly, the CD8 specificity pattern closely resembles the pattern of autoantibodies found in T1D patients and in individuals at risk for developing the disease. The autoantibodies that are routinely tested using internationally validated assays include reactivities against insulin (IAA—insulin autoantibodies), IA-2 autoantibodies (IA-2A; ICA512), GAD autoantibodies (GADA) and zinc transporter8 autoantibodies (ZnT8A) [1, 2]. Whether the similarity in reactivity pattern with that found in CD8+ islet T cells indicates related pathogenetic pathways or is an indication that both phenomena are a consequence rather than a cause of beta cell death, with the secretory-granules having a strong immunogenic effect due to their particulate nature, is open to discussion. Immunophenotyping studies in preclinical lesions [37] will be of particular importance in this context.

### Insulitis in the preclinical phase

The lesions we observe in recent-onset T1D are probably the result of a process that in many cases has been active for a considerable period of time. Diabetes registry studies, especially in first-degree relatives of T1D patients, have shown that autoantibodies against islet cell antigens can be present many years before clinical disease. They confer a risk that depends on the number of autoantibody specificities being present [55]. Autoantibodies tend to appear sequentially, with IAA appearing first, followed by GADA, IA-2A and ZnT8A [56]. They can occur over a wide age range and positivity can be transient, although only persistent positivity appears to be associated with a high risk of progression to T1D. Development of IA-2A and/or ZnT8A confer a high and age-independent risk of approximately 50 % of developing the disease within a 5-year period.

Studies on autoantibody-positive non-diabetic organ donors surprisingly showed only limited evidence of islet lesions and beta cell damage [29, 37, 57–59]. The cumulative data from these studies show that only two out of the 72 autoantibody-positive subjects showed diabetes-related histopathological changes [37]. In both subjects, <10 % of the islets were affected with insulitis; occasional pseudoatrophic islets devoid of beta cells were seen and relative beta cell area was in the normal to high normal range. Both cases presented with multiple autoantibodies in combination with a susceptible HLA-DQ genotype. Interestingly, one subject showed a considerable level of beta cell replication that was limited to the T-cell-infiltrated islets. Together, these results raise questions about our current view of the natural history of the disease and the time course of beta cell loss. They do not support a model where autoantibody positivity is a surrogate marker for extensive beta cell damage and a decreasing beta cell mass.

### Insulitis is frequent in young patients

As outlined above, insulitis is only found in a relatively small subset of diabetic patients. When the histopathological data from all population-based studies are combined [7, 19, 23, 27, 29, 32, 38, 56, 60, 61], a total of 247 cases can be analysed and stratified according to age at onset and duration of disease. Insulitis was present in 19 % of all cases. It was most prominent in young patients (0–14 years) in the first year after diagnosis: 32 of the 47 patients (68 %) in this age group showed clear insulitic lesions, albeit in often a small subset of islets. In contrast, the lesion was rare in young patients with chronic disease (>1 year), where it was only found in 4 % of cases (Table 1). The study by Foulis [24] was not included in this meta-analysis as the paper does not give clinical data on the insulitis-negative patients. However, the overall outcome of this study, with 39/45 of young patients with a disease duration of <1 year (87 %) having insulitis, is in line with the current analysis. Interestingly, insulitis was less frequently found in older individuals (15–39 years), where only 29 % of patients displayed insulitic lesions in the first year after diagnosis. The relatively low frequency in (young) adults is of interest, as it might indicate a different or less fulminant version of the disease with a slower progression of beta cell loss [9, 62].

**Table 1:** Fraction of T1D patients with insulitis stratified according to age at onset and duration of disease

	Duration of disease		
Age at onset (years)	≤1 year	>1 year	Total
0–14	32/47 (68)	4/103 (4)	36/150 (24)
15–39	10/35** (29)	2/62 ns (3)	12/97* (12)
Total	42/82 (51)	6/165 (4)	48/247 (19)

Data are expressed as number of patients with insulitis versus total number in group (percentage). Combined patient data from population-based studies [7, 19, 23, 27, 29, 32, 38, 56, 60, 61].

*ns* non-significant

*p < 0.01; **p < 0.05, significance of differences versus the age group 0–14 years was calculated using a chi-square test

### The 2013 consensus guideline for the diagnosis of insulitis

Early pathologists described insulitis as an islet-specific infiltration of a predominantly lymphocytic nature. They stressed that the infiltrates were easy to miss in conventional stains and that the lesion was probably underdiagnosed [20]. The advent of immunohistochemistry allowed phenotyping of the infiltrating cells and facilitated their quantification. However, no consensus existed in the pathology community as to which leucocyte marker should be used or how to distinguish insulitis from background infiltration. Different thresholds of 2, 3, 4, 5, 6 or 15 CD3- or CD45-positive islet cells were used by different authors to identify insulitis [7, 16, 21, 27, 29, 33, 37]. Recently, a JDRF-nPOD working group [63] defined the consensus criteria for the diagnosis of classical insulitis: ‘Patients with insulitis are defined by the presence of a predominantly lymphocytic infiltration specifically targeting the islets of Langerhans. The infiltrating cells may be found in the islet periphery (peri-insulitis), often showing a characteristic tight focal aggregation at one pole of the islet that is in direct contact with the peripheral islet cells. The infiltrate may also be diffuse and present throughout the islet parenchyma (intra-insulitis). The lesion mainly affects islets containing insulin-positive cells and is always accompanied by the presence of (pseudo)atrophic islets devoid of beta cells. The fraction of infiltrated islets is generally low (<10 % of islet profiles). The lesion should be established in a minimum of three islets, with a threshold level of ≥15 CD45+ cells/islet before the diagnosis can be made’.

### Normal islets are frequent after clinical onset

The pancreas of most recent-onset T1D patients still contains a sizeable number of insulin-positive islets: in young patients (0–14 years), approximately 38 % of islets are insulin-positive up to 1 year after diagnosis. In young adults (15–39 years), this fraction is higher with 56 % of islets being insulin positive (Table 2). These relatively high levels of residual beta cells contrast with the situation >1 year after diagnosis, where the fraction of insulin-positive islets is down to 13 %. Although these data refer to insulin-positivity at the islet level, and not to absolute beta cell mass, they do support the notion that an important residual number of beta cells are present in the first year after diagnosis. This residual beta cell mass appears to be most pronounced in individuals that develop the disease above age 14 and supports the view that the disease is less fulminant in older individuals. The quantitative analysis is supported by histopathological observations in recent-onset cases, where normal insulin-containing islets, free from insulitic infiltration, were reported to be relatively frequent [19, 40, 42]. It is also in line with an analysis of recent-onset patients, where clamp-derived second phase C-peptide release was 10–40 % of that of healthy controls [15, 56]. A recent study into the evolution of beta cell function in 948 autoantibody-positive patients <20 years who were followed a median period of 8 months after diagnosis showed a progressive decline of 4 % per month independent of age, genetic susceptibility or BMI [64]. A similar study in a demographically more heterogeneous population showed that 93 % of patients remained C-peptide positive during the 2 years follow-up period [65]. Together, these data point to a situation where many recent-onset patients have a substantial beta cell mass at diagnosis, and that this mass is decreasing relatively slowly in the months and years thereafter. The initial months after diagnosis therefore form a clear window of opportunity for studies aiming to halt or slow beta cell loss. Limiting the loss is important, as even a modest residual beta cell function will strongly benefit the patient from a clinical point of view.

**Table 2:** Percent islets with residual beta cells in T1D stratified according to age at onset and duration of disease

	Duration of disease		
Age at onset (years)	≤1 year	>1 year	Total
0–14	37.9 ± 4.1 (43)	13.5 ± 6.5* (13)	32.3 ± 3.7 (56)
15–39	56.4 ± 7.5 (11)	13.6 (1)	52.9 ± 7.7 (12)
Total	41.7 ± 3.7 (54)	13.5 ± 5.5* (14)	35.9 ± 3.4 (68)

Data are expressed as the percent of islets that contain insulin positivity (±SEM) as measured in (*n*) patients. Combined patient data from Refs. [24, 32]

*p < 0.01, significance of differences versus the group with ≤1 year duration was calculated using a non-parametric Mann Whitney test

Why many islets in recent-onset patients escape immune destruction while others become pseudoatrophic is not clear. One aspect that warrants careful analysis is the observation in several patients that the disease appears to be (multi) focal, with insulitis and pseudoatrophic islets only found in some ‘lobules’ of the gland, but not in others. It can be speculated that this is due to the anatomical structure of the gland with its multiple lobes, differences in ontogeny between ventrally derived and dorsally derived parts of the gland, structure of the ductal tree, innervation and vasculature [6].

### Beta cells persist in patients with chronic T1D

Patients with chronic (>1 year) T1D show a variable number of residual beta cells. Immunohistochemistry shows that on average 13 % of islets still contain insulin (Table 2). The remaining islets are pseudoatrophic and consist mainly of alpha cells (A cells), in addition to somatostatin-containg cells (D cells). In islets in the ventrally derived pancreatic lobe, the pseudoatrophic islets are mainly composed of pancreatic polypeptide positive cells (PP cells) in addition to D cells.

The diffuse nature of the endocrine pancreas, the heterogeneous ontogenic origin of pancreatic lobes and differences in islet composition and the focal distribution of insulitis require that extensive sampling is carried out to evaluate residual beta cell mass in patients. Very few studies have systematically sampled the whole pancreas in order to arrive at a morphometric assessment of absolute beta cell mass: one study on four T1D patients with a disease duration of 1–34 years showed a virtual disappearance of beta cells, with mean beta cell mass going from 800 to 900 mg in controls to virtually undetectable levels (<20 mg) in chronic T1D patients, but with no changes in the absolute A, D and PP cell mass [66]. A second study on two T1D patients with a disease duration of 3 and 20 years, respectively, showed a virtual absence of beta cells in the first case and a reduction to a level of 25 % of the lowest normal control in the second patient [67]. A more qualitative study of 26 T1D patients with disease duration between 2 and 54 years showed residual beta cells in 50 % of the cases. Interestingly, these cells were often found to be focally distributed and present in only a limited number of lobules. No significant relation was found between the age of onset of T1D and the presence of residual insulin positivity [68].

Several subsequent studies have confirmed the presence of residual beta cells in chronic T1D patients, even up to 84 years after diagnosis [69]. The percentage of cases with residual beta cells was estimated to be between 50 and 100 % [29, 68–70]. When only childhood (≤18 years) onset cases were included, residual beta cells were found in 6/20 chronic T1D cases [33]. The patients with residual beta cells presented with two different types of beta cell localization: in pattern A, patients showed a mixture of both insulin-deficient and insulin-containing islets, with the latter showing a clustered distribution in specific lobules of the gland. In pattern B, all islets were still insulin-containing and no insulin-deficient islets were present. A similar lobular distribution of insulin-containing islets was also observed in recent-onset patients, where insulin-containing hyperplastic islets showing insulitis were found in a limited number of pancreatic lobules, while the remainder of the gland contained only insulin-negative islets without leucocytic infiltration [19].

Patients with chronic T1D show evidence of sustained beta cell apoptosis [65, 66], possibly suggesting that in patients with chronic T1D there is a continuous formation of new beta cells and that these cells subsequently go into apoptosis due to recurrent autoimmunity or other causes. The statement in one of the early papers [68] on residual beta cells in chronic T1D patients that ‘most patients whose pancreata still contain insulin cells after a diabetes duration of 10 to 20 years, will retain these insulin cells for the rest of their lives’ should therefore perhaps be modified to allow for the possibility that the residual beta cells are in fact newly formed cells that have escaped apoptosis.

## Insulitis in the NOD mouse

The NOD mouse model was developed more than 30 years ago by Makino and Tochino while searching for a cataract-prone sub-line [71]. The strain is characterized by a high incidence of diabetes in females (approximately 80 % diabetic at week 25) and a more slowly developing phenotype in males with a lower overall incidence of diabetes [72]. Diabetogenesis is caused by multiple immunodeficiencies under polygenic control with strong environmental influences that (negatively) affect disease penetration. In addition to diabetes, the NOD mouse is prone to develop other autoimmune diseases including sialadenitis and thyroiditis. Older animals develop neoplasms, including lymphomas [73]. Genetic studies have indicated that diabetes susceptibility in the NOD mouse is linked to more than 30 different loci on 15 chromosomes [74].

### Stages of diabetes development in the NOD mouse

The pathogenesis and histopathology of the lesions in the female NOD islets of Langerhans can be arbitrarily divided into four stages (Table 3):

**Table 3:** Pathogenesis of diabetes in female NOD mice

	Age (weeks)	Events
Stage 1	4–7	Early infiltration
Stage 2	8–11	Development of inflammation
Stage 3	12–18	Development of cytotoxicity
Stage 4	>18	Clinical diabetes

### Stage 1: early infiltration (4–7 weeks)

In the NOD mouse, insulitis develops gradually, with discrete appearance of low numbers of intra-islet CD4 T-lymphocytes and their co-localization with CD11c + islet antigen presenting cells (APCs) in approximately 10 % of islets at week 4 [75]. It has been suggested in some studies that this phase is preceded by myeloid cells infiltrating the NOD mouse islet [76], while others stress that such cells are normally present in all mouse strains, independent of disease susceptibility. At week 6, approximately 30 % of islets show low level of CD4+ cell infiltration, with a median number of three cells per islet (range 1 to 55 cells per whole islet). Low numbers of CD8+ cells and B lymphocytes are present. Blood vessels inside the islet show increased ICAM-1 expression, and approximately 10 % show VCAM-1 expression. IgG deposits are found on beta cells. Gene expression profiles suggest upregulation of immune response genes, including type I interferon-inducible genes [75].

### Stage 2: development of inflammation (8–11 weeks)

At week 8, T-cell activation markers are present and the islet gene expression pattern is changed. The total number of leucocytes increases seven-fold from 2.7 to 18 % of islet cells as determined by FACS in dissociated islets [75]. There is infiltration of 50–60 % of islets with all major inflammatory cell types, including CD4, CD8 and CD11c cells and in some islets B cells. The infiltrating cells are preferentially located in the islet periphery and are sometimes focal at one pole of the islet periphery. Infiltration is not homogeneously distributed throughout the gland, but appears to be localized, with some pancreatic areas being affected and others not [77]. Islets show an increased endothelial expression of ICAM-1 and VCAM-1 [75]. Beta cells show increased expression of MHC class I [78]. Sizeable focal accumulations of CD3+ lymphocytes are being formed, most of which are in close contact to islet tissue. Part of these clusters (15 % at week 8, increasing to 81 % at week 20) resemble tertiary lymphoid organs (TLO), with separated T- and B-cell compartments, specialized vasculature including lymphatic vessels and high endothelial venules. The T-cell zone usually lies closest to the islet and is capped by a more distal B-cell zone. TLOs have been described in many chronic inflammatory diseases (but not human diabetes) and are thought to play a role in disease progression [77, 79–81]. Total beta cell volume in the NOD pancreas does not change significantly between 6 and 12 weeks of age [77], indicating that the predominantly peri-insulitis at this stage is of a non-destructive nature.

### Stage 3: cytotoxicity develops (12–18 weeks)

Between weeks 12 and 18, there is a shift towards a cytotoxicity-related gene expression pattern together with an overall increase in the number of infiltrating cells [75]. At week 17, 58 % of islets are found to show peri-insulitis with a mixture of lymphocytes and monocyte-derived cells completely surrounding the islet and 5 % of islets showing intra-insulitis, associated with the erosion of beta cells mass and with only some residual beta cells remaining. At week 18, all islets are affected by infiltrates, with CD45+ cells forming approximately 40 % of the islet cells. Total beta cell mass gradually decreases, with a 42 % reduction at week 13 and a 73 % reduction at week 18 versus age-matched animals of a related control strain [77, 82].

### Stage 4: clinical diabetes (>18 weeks)

Despite a prolonged inflammatory phase starting at week 8, most female mice only become diabetic after weeks 18–20 [54]. At clinical onset, total beta cell volume is decreased to approximately 2.7 10^9^ μm^3^, which is a 86 % decrease compared to that found in 16-week-old diabetes-resistant control mice [2]. In diabetic animals, the pseudoatrophic islets are devoid of beta cells and are free of inflammatory infiltrates.

## Comparing insulitis in human disease with the NOD mouse model

### The histopathology of insulitis in the NOD mouse is different from that in patients

**Fig. 1:**
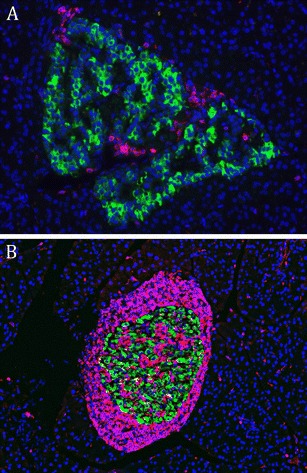
**a**. Insulitis in a patient with T1D showing infiltrating CD45+ leucocytes (*red*) in an insulin-positive islet of Langerhans (*green*) (×600). **b**. Insulitis in a 20-week-old female NOD mouse showing infiltrating CD3+ T cells in an insulin-positive islet (green) (×300)

When insulitis in recent-onset human T1D is compared to the lesions found in the NOD mouse, some major differences can be observed (Table 4). The human disease is characterized by a relatively mild infiltration (Fig. 1a) that only affects a small fraction of the islets. Quantification of the infiltrates [75] shows an average of 25–30 CD8+ T cells per islet section, which is one or more orders of magnitude lower than the massive infiltration observed in the NOD mouse (Fig. 1b). In the mouse model, the infiltration starts with a peri-islet accumulation of CD3+ T cells, often encompassing the whole islet and increasingly showing the spatial organisation of a tertiary lymphoid organ. Neither islet encompassing peri-insulitis nor TLOs have been described in the literature for the human disease. A fourth major difference between insulitis in T1D patients and NOD mice is the fraction of islets that is affected. In 18 week-old, female NOD mice virtually all islets are affected by a marked lymphocytic infiltration. Information on the histopathology of patients in a preclinical phase of the disease is limited, as only a small number of autoantibody-positive non-diabetic cases with insulitis have been described [37]. In these cases, inflammation was limited to <10 % of islets. This is similar to the situation in a group of 54 young recent-onset patients (<1 year duration), where insulitis was present in an average of 9 % of all islets (range 0–35 %). When only islets were analysed that still contained beta cells, an average of 32 % of such islets showed insulitis [24, 32].

**Table 4:** Comparing insulitis in female NOD mice and patients

Histopathological characteristics	Human	NOD
Insulitis present at onset	Yes, low level	Yes, massive
Infiltrate predominantly T cells	Yes	Yes
Infiltrate contains autoreactive CD8+ T cells	Yes	Yes
Beta cell mass reduced at diagnosis (% of normal)	20–30 %	10–20 %
Massive encircling peri-insulitis prior to and at onset.	No	Yes
Peri-insulitis with tertiary lymphoid organs present at onset	No	Yes
HLA class I hyperexpression	Yes	Yes

### Immunophenotyping of insulitis shows similarities

Having stressed the morphological differences, it is also important to highlight some of the (immunological) similarities: insulitis in both species contain a high fraction of T cells, especially of the CD8 type, in addition to macrophages and B lymphocytes. The specificities of the autoreactive islet-infiltrating CD8 cells in both species include insulin and IGRP epitopes [74]. The infiltrates in the NOD start at 4–7 weeks, shortly before the female becomes fertile around 8 weeks of age, they become diabetic some 10–20 weeks later. In patients, diabetes incidence peaks around puberty, thus appearing to predate the disease in the NOD to a minor degree. Both show insulitis most prominently in the beta cell containing islets, while pseudoatrophic islets are virtually devoid of infiltrating cells. Both also show a marked overexpression of class I MHC in endocrine cells [78]. In patients, MHC class I hyperexpression was prominent in recent-onset cases [29, 32] and was also found in pseudoatrophic islets devoid of insulin positivity [29].

### Early stages of insulitis are associated with beta cell replication

Observations in the NOD mouse pancreas support a model in which inflammatory infiltration stimulates replication. Beta cell replication was found to increase in pre-diabetic mice [82] and after adoptive transfer of diabetogenic spleen cells [83]. The increase in replication was found to precede a significant decline in beta cell mass and coincides with the early phase of insulitis, suggesting that inflammation associated cytokines may mediate the effect. Recent studies in NOD.RAG1^−/−^ mice transferred with total diabetic splenocytes reported a linear correlation between the islets manifesting insulitis and beta cell proliferation [84], although this somewhat contrasts with early observations that mitotic figures in islet beta cells are preferentially found in islets that are not yet affected by insulitis [85]. Some of the observations found in the NOD mouse may also be present in patient material: a 59-year-old autoantibody-positive organ donor without a clinical history of diabetes, but with a susceptible HLA-DQ genotype and positivity for four different autoantibodies, showed a marked Ki67-positivity in islets with ongoing insulitis [37]. The replication marker localized with both insulin and glucagon positivity, suggesting that the inflammatory environment was stimulating replication of all islet cell types. Similar findings were reported for autoantibody-negative organ donors where organs with a high level of diffuse pancreatic leucocytic infiltration were also found to present with high levels of islet cell replication [86]. Increased levels of replication were also found in ten patients with recent-onset T1D [87], but could not be confirmed in another cohort of nine recent-onset patients who died of ketoacidosis [27].

## Conclusion

From a histopathological point of view, the insulitic lesions in the islet of Langerhans of recent-onset T1D patients are very different from those observed in the NOD mouse. In the animal model, the infiltrate is massive, affects all islets and has a clear peri-islet phase that precedes the development of beta cell cytotoxicity. None of these characteristics are observed in patients, where the infiltration is relatively subtle in terms of the numbers of infiltrating cells, is only observed in part of the islets and is not observed in all cases. Arguably, the disease in the NOD mouse also has many pathogenetic elements in common with the human disease: the genetic predisposition, polygenic trait and contribution of the MHC-loci, together with a strong environmental influence, are well known in both patients and NOD mice. Importantly, both appear to share a common effector mechanism, with CD8+ T-cell autoreactivity against known beta cell components. However, in view of our limited data on the human disease, care should be taken when using the observations in an autoimmune rodent model that resembles some, but not all, aspects of T1D, to make predictions and devise immune therapies in patients. A recent thoughtful review [88] listed the arguments pro- and contra the assertion that human T1D is a T-cell-mediated autoimmune disease. The authors argue ‘that the presence of T cells in or around islets is not a general finding in T1D; that the process of tissue injury in T1D is not as specific as would be expected for a T-cell-mediated autoimmune disease and that the progression of T1D is generally slow and the loss of beta cells is heterogeneous throughout the pancreas, contradictory to what would be expected if the disease was mediated by a T-cell response against islet antigens’, instead they offer an alternative hypothesis that an influx of different bacterial species from the duodenum via the papilla of Vater into the pancreatic ductal tree leads to the activation of ductal epithelial cells and islet cells, innate periductal inflammation, loss of exocrine parenchyma and periductal fibrosis. The periductal inflammation would be particularly harmful to the beta cells due to their low protection against NO, ROS and proinflammatory cytokines. The lobular nature of such a (repeated) innate inflammatory process could explain the heterogeneous nature of the disease in different pancreatic lobes [89]. Similarly, others have argued in favour of a viral aetiology of the disease with many different types of viruses, including rubella virus, cytomegaloviris, mumps virus and enterovirus linked to the development of T1D [90]. The most striking case being that of a 10-year-old boy who developed T1D and where coxsackie B4 virus was isolated from the pancreas and used to inoculate mice leading to insulitis and beta cell death in these animals [53].

The pioneer of islet histopathology, Philip Lecompte, proposed four explanations for his 1958 observation of insulitis in children [20], namely a direct invasion of the islets by an infectious agent, a manifestation of functional overstimulation or strain, a reaction to damage by some unknown nonbacterial agent and antigen-antibody reaction. We should conclude that these different explanations are all still relevant today and that the discussion about the aetiology of the disease is still open.
